# Daily online contouring and re-planning versus translation-only correction in neurovascular-sparing magnetic resonance-guided radiotherapy for localized prostate cancer

**DOI:** 10.1016/j.phro.2022.09.002

**Published:** 2022-09-13

**Authors:** Frederik R. Teunissen, Jochem R.N. van der Voort van Zyp, Eline N. de Groot-van Breugel, Helena M. Verkooijen, Ruud C. Wortel, Johannes C.J. de Boer

**Affiliations:** a Department of Radiation Oncology, University Medical Center Utrecht, the Netherlands; b Imaging and Oncology Division, University Medical Center Utrecht, the Netherlands; c Utrecht University, the Netherlands; d Department of Urology, University Medical Center Utrecht, the Netherlands

**Keywords:** MRgRT, Localized prostate cancer, MR-Linac, Neurovascular-sparing, Adapt-to-shape, Adapt-to-position

## Abstract

Neurovascular bundle (NVB) and internal pudendal artery (IPA) sparing during magnetic resonance-guided radiotherapy (MRgRT) for prostate cancer aims for preservation of erectile function. Our present workflow involves daily online contouring and re-planning on a 1.5 T MR-linac, as alternative to conventional (rigid) translation-only corrections of the prostate. We compared planned dose for the NVB and IPA between strategies. Total planned dose was significantly lower with daily online contouring and re-planning for the NVB, but not for the IPA. For the NVB and IPA, the intrapatient difference between highest and lowest fraction dose was significantly smaller for the contouring and re-planning plans.

## 1 Introduction

Erectile dysfunction after stereotactic body radiation therapy for prostate cancer (PCa) occurs in 26% to 55% of patients five years after treatment [1]. Literature suggests that radiation damage to predominantly the neurovascular bundle (NVB), internal pudendal artery (IPA), corpus cavernosum (CC), and penile bulb (PB) causes erectile dysfunction [2].

Our group previously demonstrated that neurovascular-sparing magnetic resonance-guided adaptive radiotherapy (MRgRT), sparing the NVB, IPA, CC, and PB, is feasible. Currently, the first trial investigates this treatment's clinical outcomes in the single-arm phase-II ERECT trial (NCT04861194) [3], [4]. In the trial setting, patients with intermediate-risk PCa and sufficient to good erectile function at baseline (i.e., IIEF-5 score of ≥ 17) are treated [5].

MRgRT enables online 1.5 Tesla (T) MR imaging before and during every fraction. Because soft tissue can be visualized with diagnostic quality, online contouring and re-planning can be performed, also called “adapt-to-shape” (ATS) [6]. This procedure is a step further than online (rigid) translation-only correction in x, y, and z directions based on the prostate location, or “adapt-to-position” (ATP). Online translation-only correction is generally applied on conventional CT-guided radiotherapy devices using fiducial markers in the prostate. Because fiducial markers give information about the position of the prostate but not of the exact shape and due to the lack of soft tissue contrast on CT imaging, online contouring and re-planning for the NVBs may not be adequately applied on conventional external beam radiotherapy (EBRT) [2], [7].

The position and shape of soft tissue structures may change under the influence of bladder and rectum filling between and during fractions. Therefore, daily online contouring and re-planning may have an advantage in both dose coverage of the target volumes and dose sparing of the OARs, including the neurovascular structures [8].

The CC and PB are generally located more distant from the prostate than the NVB and IPA. The dose reduction of neurovascular-sparing MRgRT is, therefore, predominantly accomplished in the NVB and IPA compared to standard MRgRT and the hypothetical advantage of daily online contouring and re-planning over translation-only correction is most relevant in those structures [3].

In this paper, we assess the dose/volume-based difference of daily online contouring and re-planning versus translation-only correction for the NVB and IPA in neurovascular-sparing MRgRT for localized PCa.

## 2 Materials and methods

### 2.1 Patients and treatment

The first 20% (14/70) of patients treated within the ERECT trial were included. The ERECT trial received approval from the Institutional Review and Ethics Board of the University Medical Center Utrecht, the Netherlands. Patients signed informed consent for sharing of their data. All patients received neurovascular-sparing MRgRT of 36.25 Gy in five fractions on a 1.5 T MR-Linac (Unity, Elekta AB, Stockholm, Sweden). Prior to radiotherapy, an offline 3 T MRI was made on which all structures were contoured (i.e., target volumes and OAR) using the in-house developed software tool “Volumetool”. This planning MRI including contour set was imported into the treatment planning software to generate intensity modulated radiation therapy (IMRT) offline treatment plans. The gross tumor volume (GTV) + 4 mm consisted of the MR-visible tumor with a 4 mm isotropic margin excluding the rectum and bladder. The clinical target volume (CTV) included the GTV + 4 mm and prostate body with the base of the seminal vesicles, and the planning target volume (PTV) included the CTV with a 5 mm isotropic margin. Depending on the position of the GTV, bilateral, unilateral, or no NVB sparing was utilized, which was determined prior to pre-treatment planning to ensure sufficient homogeneous GTV and PTV coverage during planning, and only allow a minor PTV dose reduction adjacent to the spared NVB. IPA, CC, and PB sparing was always utilized. Dose prescriptions were 34.4 Gy (95%) in ≥ 99% for the GTV + 4 mm isotropic margin (excluding rectum and bladder) and the 30.0 Gy in ≥ 99%; 32.6 (90%) Gy in ≥ 90% for the PTV. In the case of bilateral NVB sparing, the PTV 34.4 Gy dose prescription was set at ≥ 80%, in the case of unilateral NVB sparing ≥ 90%, and in the case of no NVB sparing ≥ 99%. This was done because for the unilateral and no NVB-sparing setting, the higher PTV 34.4 Gy dose prescription did not compromise the sparing of any of the other neurovascular structures or conventional OAR but resulted in a higher PTV coverage [3]. The dose constraint for the NVB was D0.1 cc ≤ 32.8 Gy, and for the IPA D0.1 cc ≤ 20.0 Gy. The IPA constraint was based on a previous vessel-sparing trial [9], and the NVB constraint on the literature on neural and vascular tissue and experience with radiation toxicity of the sacral plexus and brachial plexus (all dose prescriptions and constraints in supplementary materials) [3]. During treatment planning and online plan adaptation, we used a template in which target coverage was the primary goal, meeting the constraints of the conventional OAR the secondary goal, and meeting the neurovascular constraints the tertiary goal. Furthermore, the NVB constraint was a soft constraint.

**Fig. 1:**
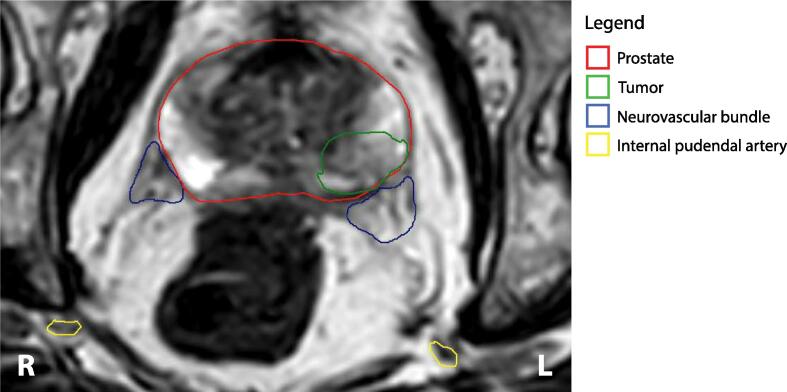
Axial representation of the re-contoured prostate, tumor, neurovascular bundles, and internal pudendal arteries on an online 1.5 T T2w MR scan (patient 14; fraction 1). Abbreviations: L = left; R = right.

During every fraction, an online 1.5 T T2w MR scan was made on which the pre-treatment contours were registered using a semi-automated deformable registration method (sequence parameters in supplementary materials). The contours of the target volumes and OAR were automatically adapted to shape and if needed, adjusted manually (Fig. 1). This process was generally accurate for high-contrast structures on T2-weighted MRI, such as bladder, rectum, and IPA. Lower-contrast structures such as the prostate and especially the NVB generally had to be adjusted manually. In the next step, the treatment plan was adapted to the anatomy of the day. Dose to the NVB and IPA are controlled by cost functions. Isoconstraints of the cost functions were adjusted during online planning for plan optimization, but no cost functions were added or removed. During the treatment plan calculation, an online 3D T2w position verification MR scan was made. The position verification scan was used to perform an additional ATP procedure directly before beam on to account for intratreatment patient motion during the ATS procedure [10], [11].

### 2.2 Planned ATS and simulated ATP dose

All planning was done in Monaco 5.40.01. To compare the planned dose to the NVB and IPA in the ATS setting to the planned dose which would have been received in the ATP-only setting, the ATP dose was simulated. Therefore, the daily planning MR scan of each fraction was matched (translations only) to the pre-treatment scan based on the prostate contour. The adapted daily contours of the NVB and IPA of each fraction were copied to the structure set on the pre-treatment scan using the rigid registration obtained from the prostate match. The planned dose that was received by the NVB and IPA of each fraction in case the pre-treatment plan would have been delivered for each fraction and only corrected for prostate translation as would have been done in an ATP-only workflow was calculated (i.e., simulated ATP planned dose).

The ATS planned dose to the NVB and IPA was calculated based on the plan that was calculated using the online pre-treatments scan after ATS was performed but before the position verification using ATP was performed. The used contours were identical to the simulated ATP contours. Planned ATS and simulated ATP dose-volume histograms for the NVB and IPA were exported.

### 2.3 Statistical analysis

R version 4.1.2 was used for the statistical analysis. The maximum dose to the 0.1 cc (D0.1 cc) of the NVB and IPA for each fraction of each patient was calculated using the R package “dvhmetrics”. Per patient, estimated mean total dose and width of variance (difference in Gy between lowest and highest fraction multiplied by 5) for the planned ATS and simulated planned ATP setting were calculated. Pairwise comparisons of total dose and width of variance between the planned ATS and simulated planned ATP were analyzed using Wilcoxon signed-rank tests. Non-normally distributed data were presented as median with interquartile range (IQR). A p-value of < 0.05 was considered statistically significant.

## 3 Results

Fourteen patients with intermediate-risk prostate cancer completed treatment within the ERECT trial and were included. Bilateral NVB sparing was accomplished in three (21%) patients, unilateral NVB sparing in nine (64%), and no NVB sparing in two (14%) patients. IPA sparing was accomplished in all patients.

**Fig. 2:**
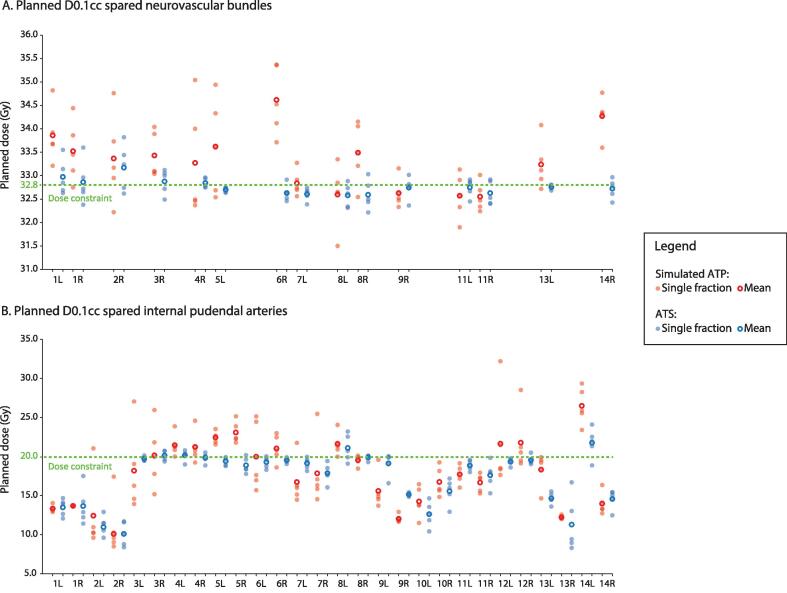
Planned D0.1 cc for patients treated with neurovascular-sparing MRgRT for localized prostate cancer. A. Spared neurovascular bundles. B. Spared internal pudendal arteries. The single-fraction dose indicates the dose multiplied by 5. Abbreviations: L = left; R = right; ATP = adapt-to-position; ATS = adapt-to-shape.

For the NVB D0.1 cc, the median (range) total planned dose for ATS was 32.7 Gy (32.6 – 33.2) and for simulated ATP 33.4 Gy (32.6 – 34.6) (p = 0.002) (Fig. 2). The median (range) width of variance was 0.5 Gy (0.1 – 1.2) and 1.6 Gy (0.7 – 2.7) (p < 0.001), respectively. For the planned IPA, the median (range) total planned D0.1 cc for ATS was 19.0 Gy (10.1 – 21.8) and for simulated ATP 18.0 Gy (10.1 – 26.5) (p = 0.116) (Fig. 2). The median (range) width of variance was 2.2 Gy (0.6 – 8.4) and 4.4 Gy (0.1 – 14.6) (p = 0.004), respectively.

The mean NVB dose exceeded the constraint in 4/15 (26.7%) NVBs in the ATS plans and 10/15 (66.7%) NVBs in the ATP plans. For the IPA constraint, that was 4/28 (14.3%) IPAs and 10/28 (35.7%) IPAs, respectively.

## 4 Discussion

In this paper, we assessed the dose/volume-based difference between daily online contouring and re-planning versus translation-only correction for neurovascular-sparing MRgRT. An advantage of daily MR scanning is the re-evaluation of the pre-treatment contours. Factors such as scan quality and interrater variability can lead to suboptimal contours. Manual adjustment during daily online contouring and re-planning allows for re-evaluation of the pre-treatment contours, and in case of a substantial difference, it can be decided to make a new optimized pre-treatment plan for the remaining fractions. In our series, this was done in one patient (patient 12), where we adjusted the IPA contours after re-evaluation based on the online MR scan and made a new optimized pre-treatment plan upon after the second fraction.

The current drawback of online contouring and re-planning is that the contours need to be adjusted manually. This process takes time, during which there can be continuing motion and deformation. Therefore, a subsequent position verification MR scan and translation-only correction is performed. When becoming available, fast online auto contouring and plan adaptation before beam-on will make the manual adjustment of contours obsolete and reduce the need for subsequent position verification for intrafraction motion [12], [13], [14].

The question we wanted to answer in this study was: how does a daily ‘perfect’ shift of a pre-determined reference dose distribution perform with respect to a daily dose re-optimization based on daily adapted contours regarding the sparing of neurovascular structures? Therefore, we chose the method described in section 2.2 (i.e., to evaluate the reference dose in the daily adapted contours after a shift based on the alignment of the prostate soft tissue). Executing such shifts depends on the specific hard- and software used in the adaptive radiotherapy setting. Whereas for conventional CT-guided radiotherapy, this is usually an actual couch shift, for the MR-Linac systems, it would be a virtual couch which may yield small deteriorations with respect to a perfect shift [6]. However, we considered such small template-dependent differences beyond the scope of this paper.

Another limitation is the inter- and intrarater variability of the contouring of the IPA and especially the NVB, which is a lower contrast soft-tissue structure with less pronounced boundaries at the level of the prostate base [15], [16], [17]. This may have led to an over- or underestimation of the difference between ATP and ATS in this study. However, our previous work showed that interrater variability is substantially lower at the mid prostate to apex level [15]. This is the level where the NVB is closest to the prostate, and any contour shifts will relatively have the largest effect on NVB dose, therefore making our ATP and ATS dose estimates more reliable. Also, in our clinical trial setting, the offline contouring is performed by a single specialized radiation oncologist and online by one of a team of four specialized radiation oncologist. One dedicated researcher supervises all contours, reducing the inter- and intrarater variability.

In conclusion, we showed that for the NVB, daily online contouring and re-planning resulted in lower median total dose compared to translation-only correction. Furthermore, the intrapatient width of variance of fraction dose for the NVB and IPA was lower with daily online contouring and re-planning. The high-field MR-Linac enables daily online contouring and re-planning for soft-tissue structures with low contrast. Our findings support the utilization of this treatment strategy and the further development of fast online auto contouring and real-time plan adaptation for optimal neurovascular-sparing treatment for localized PCa.

## Role of the funding source

This research has been partly funded by ZonMW IMDI/LSH-TKI Foundation (The Hague, The Netherlands, project number 104006004), Elekta AB (Stockholm, Sweden), and Philips Medical Systems (Best, The Netherlands). The funding sources had no involvement in the design of the study, the collection, analysis, and interpretation of the data, nor in the writing and decision to submit the article for publication.

## Declaration of Competing Interest

The authors declare the following financial interests/personal relationships which may be considered as potential competing interests: HV receives research funding from Elekta. The remaining authors declare no potential competing interests.
