# Supplementary material for: Daily online contouring and re-planning versus translation-only correction in neurovascular-sparing magnetic resonance-guided radiotherapy for localized prostate cancer
Source: Phys Imaging Radiat Oncol. 2022 Sep 13;24:43–6. doi: 10.1016/j.phro.2022.09.002 (PMC9485897; doi:10.1016/j.phro.2022.09.002)
Supplement: Supplementary Data 1 [file mmc1.docx]

**Supplementary materials**

**Table 1:** Target volume dose prescription and dose constraints for neurovascular-sparing 5 x 7.25 Gy MRgRT.

| Structure | Parameter | Dose constraint | |
| --- | --- | --- | --- |
|  |  | Soft | Hard |
| PTV | V34.4Gy (V95%) |  | ≥ 80.0% bilateral NVB sparing*  ≥ 90.0% unilateral NVB sparing*  ≥ 99.0% no NVB sparing* |
|  | V32.6Gy (V90%) |  | ≥ 90.0% |
|  | V30.0Gy (V83%) |  | ≥ 99.0% |
| GTV+4 mm | V34.4Gy (V95%) |  | ≥ 99.0% |
| Bladder | D0.5cc |  | < 42.0 Gy |
|  | D5cc |  | < 37.0 Gy |
|  | V32.0 Gy |  | < 15.0% |
|  | V28.0 Gy |  | < 20.0% |
| Femur | D10cc | < 30.0 Gy |  |
| Rectum | D0.5cc |  | ≤ 40.0 Gy |
|  | D1cc | ≤ 35.0 Gy | ≤ 38.0 Gy |
|  | V32.0 Gy |  | ≤ 15.0% |
|  | V28.0 Gy |  | ≤ 20.0% |
| Sphincter (distal 3 cm of rectum) | D0.5cc |  | ≤ 40.0 Gy |
|  | D1cc | ≤ 35.0 Gy | ≤ 38.0 Gy |
|  | Dmean |  | < 20.0 Gy |
| NVB | D0.1cc | ≤ 32.8 Gy |  |
| IPA | D0.1cc |  | ≤ 20.0 Gy |
| CC | D0.01cc |  | ≤ 17.3 Gy |
| PB | D50% |  | < 29.5 Gy |

*Depending on the position of the GTV in relation to the NVBs

Abbreviations: GTV = gross tumor volume; PTV = planning target volume; NVB = neurovascular bundle; IPA = internal pudendal artery; CC = corpus cavernosum; PB = penile bulb.

**Table 2**

3D T2-weigted TSE MRI sequence parameters.

| FOV (mm) | 400x446x180 |
| --- | --- |
| resolution (mm) | 0.78x0.78x2 |
| flipangle (degree) | 90, refocussing control: 100 |
| TE (ms) | 120 |
| TR (ms) | 1635 |
| Readout bandwidth (Hz/pixel) | 562 |

Abbreviations: TSE = turbo spin echo; FOV = field-of-view ; TE = echo time; TR = repetition time

Acquisition time: 3:05 minutes
